# Albany Medical College

**Published:** 1867-05

**Authors:** 


Albany Medical College.—Our advertisement sheet contains announcement of the Lecture Term in the Albany Medical College. It will be noticed that S. Oakley Vanderpoel, M. D., has been appointed Professor of General Pathology and Clinical Medicine; James E. Pomfret, M. D., Professor of Physiology; John V. Lansing, M. D., Professor of Materia Medica. These recent additions to the faculty will, no doubt, increase the advantages of the school. Its prosperous condition may be seen in the number of yearly graduates; the last term furnished fifty-three.

